# Dataset of Factors affecting online cheating by accounting students: The relevance of social factors and the fraud triangle model factors

**DOI:** 10.1016/j.dib.2021.107732

**Published:** 2021-12-18

**Authors:** Mohannad Obeid Al Shbail, Hashem Alshurafat, Husam Ananzeh, Jebreel Mohammad Al-Msiedeen

**Affiliations:** a Assistant professor, School of Business, Al al-Bayt University. P.O.BOX 130040. Mafraq 25113, Jordan; b Assistant Professor, Department of Accounting, Faculty of Economics and Administrative Sciences, Hashemite University. P.O. Box 330127, Zarqa 13133, Jordan; c Assistant Professor, Irbid National University, Irbid, Jordan; d Jebreel Mohammad Al-Msiedeen, Assistant Professor, Tafila Technical University, Jordan

## Abstract

In this paper, the authors present survey data concerning to understand the motivations underlying the cheating behavior while university exams are held online during the Covid-19 pandemic. In pursuing its aims, this study uses an integrated theoretical framework that includes the social capital theory and the fraud triangle theory. Through the use of a previously tested questionnaire, this study gathers data concerning the students cheating behavior from 213 respondents across a group of Jordanian universities. The findings of this study show that pressures, opportunities, rationalization, social norms, and social trust are all factors that affect the behavioral intention to cheat, which ultimately lead accounting students to commit cheating while taking exams online. This research provides several practical contributions to the educators who are seeking to minimize the chances for dishonest students to cheat in online exams. Future studies can refer to the study and its findings when it comes to educational equity and policy making regarding distance education.

**Specifications Table**

**Table:** 

Subject	Education
Specific subject area	e-learning system; accounting students
Type of data	Table Figure Excel file
How data was acquired	The required data was collected via online questionnaire that was introduced to the online survey platform, Microsoft Forms, converted into .csv format. The questionnaire copies contained items measured on a Likert-type scale, translated into Arabic language, which is the mother-tongue of Jordanians. A copy of the questionnaire can be accessed from the Internet using the following links; English version: https://forms.office.com/r/D172at2evF Arabic version: https://forms.office.com/r/J62QpAytaz
Data format	Raw Analyzed
Parameters for data collection	Data on behavioral intention to cheat while university exams are held online during the Covid-19 pandemic was collected from March 15, 2021, to May 15, 2021 in a two-month span, and from the distributed questionnaires, 231 were retrieved, and following a review of responses content, 18 were dropped because of lack of information/missing data, which leaves a total of 213 questionnaires suitable for statistical analysis.
Description of data collection	The online questionnaire was forwarded to different accounting instructors teaching accounting courses in the Jordanian universities, and they were requested to disseminate copies of it to the students through the questionnaire's link. The students were sampled using snowball sample technique to promote more participants as the students were encouraged to forward the link to their classmates going to the same universities.
Data source location	Region: Asia Country: Jordan
Data	Mendeley data
Data accessibility	Repository name: Mendeley data. Data identification number: 10.17632/zxc6sdsj8d.2 Direct URL: https://data.mendeley.com/datasets/zxc6sdsj8d/1

## Value of the Data


 

• Data was fully used in its description of the way opportunities, pressures, rationalization, subjective norms, social trust, behavioral intention to cheat can influence the cheating behavior among accounting students in the context of Jordan during the Covid-19 pandemic;
• The dataset is useful for further comparisons in further studies dedicated to distance learning issues among nations that have distinct contexts and are experiencing different stages of Covid-19 pandemic;
• Data is useful in providing insights and direction on the way to understand the motivations underlying the behavioral intention to cheat while university exams are held online through the integrated theoretical framework that includes the social capital theory and the fraud triangle theory based on literature;
• Dataset can be used as a guide and reference by the educators who are seeking to minimize the chances for dishonest students to cheat in online exams;
• The dataset outcome not only can help educational universities to understand how severe is the online cheating problem among students in Jordan, but they can also help them identify the underlying motives behind the cheating behavior during these difficult times. Such understanding is particularly important to help these institutions producing a more fair assessment system for students' performance while adopting the online learning system as the primary approach of education in the country.

## 1 Data Description

The study gathered data regarding the E-learning system usage within two months (March 15, 2021, to May 15, 2021), from the students hailing from different accounting faculties (accounting, accounting and commercial law, accounting and business law, accounting information system) in Jordanian universities. The main data collection instrument used is the questionnaire, where copies of it were disseminated online using Microsoft Forms. The questionnaires were retrieved from 231 students, but 18 of them were dropped because of lack of information/missing data in the responses content. The remaining 213 questionnaires were deemed valid for statistical analysis.

Before the actual data collection, the questionnaire was developed in two languages, English and Arabic, because both languages are used as the official and formal languages in the Jordanian universities for teaching accounting. The research group consisted of individuals who were experienced and fluent in English and Arabic, which discounted the requirement for an expert panel. Specifically, the research group, comprised of accounting lecturers, who had knowledge and experience in E-learning system and because the universities use similar E-learning systems, the participants need not be categorized on the basis of the universities they studied in. In the final questionnaire, the items were divided into three sections (introduction, socio-demographic information and construct measurements), with the third section containing items that measure the constructs gauged on a 5-point Likert scale. The raw data and the original questionnaire are available in Mendeley data at https://data.mendeley.com/datasets/zxc6sdsj8d/1.

Details of the respondents’ profile are presented in Table 1, and from the table, it is evident that female respondents (62.0%) outnumbered that of their male counterparts (38.0%). The majority of the study sample (84.5%) were obtaining their bachelor's degrees, while the rest (15.5%) were obtaining their master's degrees. Over half of them were in the accounting faculty (60.1%), followed by those in accounting and commercial law (32.9%), then accounting and business law (6.1%), and lastly, accounting information systems (0.9%). Moving on to their academic years, majority of the respondents were in their junior year (28.1%), followed by senior year students (25.8%), sophomore (18.8%), freshman (12.2%), senior master students in their second year (8.5%) and lastly, master students in their first year (6.6%).

**Table 1:** Respondents’ profile.

Demographic variables	Category	Frequency	(%)
Gender	Male	81	38.0
	Female	132	62.0
Program	Bachelor	180	84.5
	Master	33	15.5
Academic year	Bachelor “Freshman” (first year)	26	12.2
	Bachelor “Sophomore” (second year)	40	18.8
	Bachelor “Junior” (third year)	60	28.1
	Bachelor “Senior” (fourth year)	55	25.8
	Master (first year)	14	6.6
	Master (second year)	18	8.5
Specialization	Accounting	128	60.1
	Accounting and Commercial Law	70	32.9
	Accounting and Business Law	13	6.1
	Accounting Information Systems	2	0.9

**Fig. 1:**
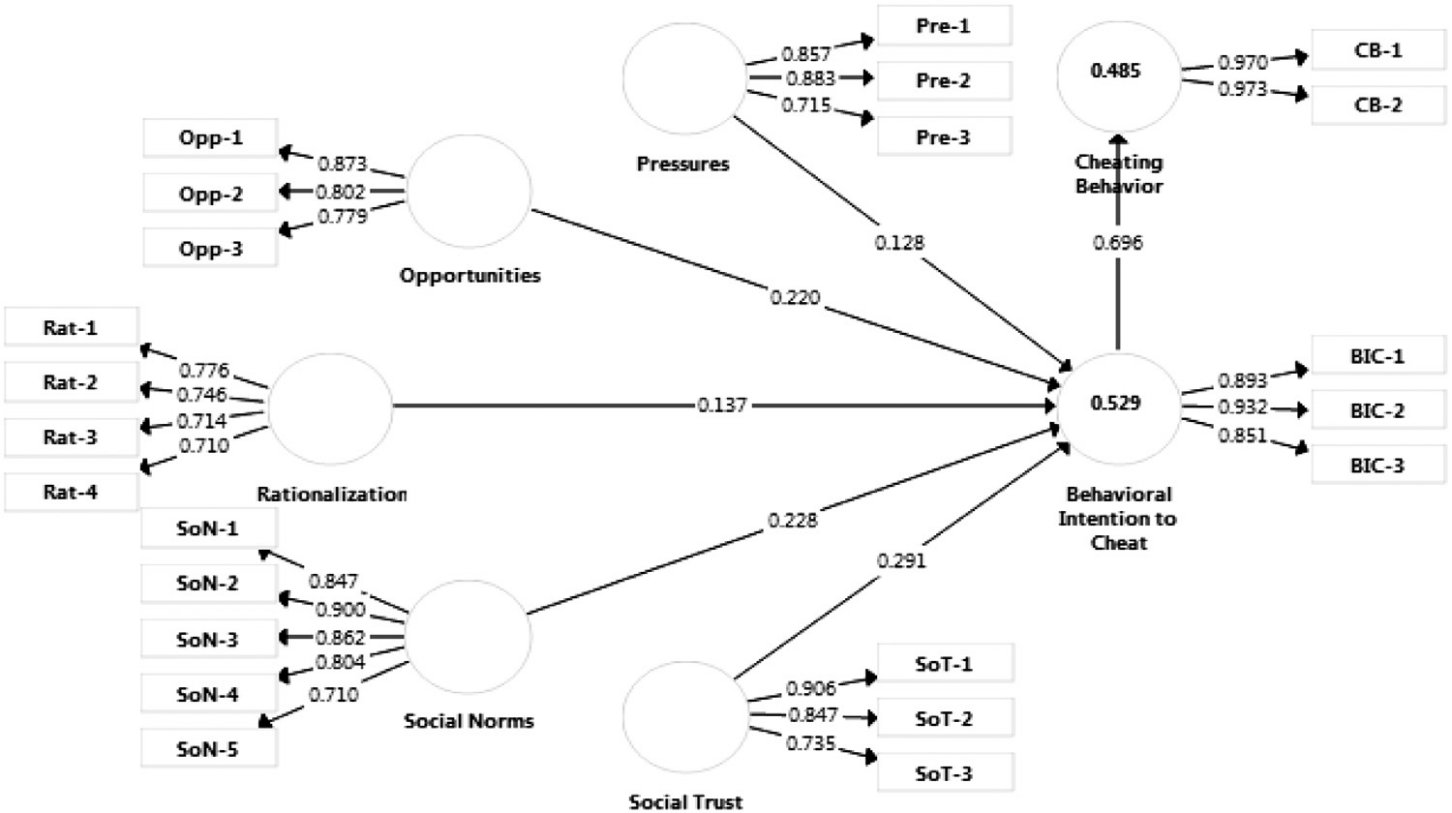
Measurement model results.

The measurement or outer model was evaluated through the observation of the factor loadings of individual measurement items, their Cronbach's alpha values and Composite Reliability (CR) values, as well as, Average Variance Extracted (AVE) to establish the presence of convergent validity [1]. With regards to discriminant validity, it was confirmed using the criterion proposed by Fornell and Larcker [2] and Heterotrait and Monotrait (HTMT) ratio as recommended in literature [3], [4], [5]. The analysis results are presented in Table 2, and from the table, a good level of internal consistency was found, supported by the Cronbach's alpha values and CR values that were over 0.71. This meets the rule of thumb established by [6]. In addition, the values of individual item loadings also exceeded 0.70 and were significant at (*p* < 0.001), and AVE values of the constructs exceeded the established threshold of 0.50 (see Table 2 and Fig. 1 for details), which shows that the model had convergent validity [1].

**Table 2:** Measurement model results.

Construct		Code	Loadings	Cronbach's α	CR	AVE
**Pressures**		Pre-1	0.857	**0.762**	**0.861**	**0.675**
		Pre-2	0.883			
		Pre-3	0.715			
**Opportunities**		Opp-1	0.873	**0.754**	**0.859**	**0.671**
		Opp-2	0.802			
		Opp-3	0.779			
**Rationalization**		Rat-1	0.776	**0.721**	**0.826**	**0.543**
		Rat-2	0.746			
		Rat-3	0.714			
		Rat-4	0.710			
**Social Norms**		SoN-1	0.847	**0.884**	**0.915**	**0.684**
		SoN-2	0.900			
		SoN-3	0.862			
		SoN-4	0.804			
		SoN-5	0.710			
**Social Trust**		SoT-1	0.906	**0.779**	**0.870**	**0.693**
		SoT-2	0.847			
		SoT-3	0.735			
**Behavioral Intention to Cheat**		BIC-1	0.893	**0.872**	**0.921**	**0.796**
		BIC-2	0.932			
		BIC-3	0.851			
**Cheating Behavior**		CB-1	0.970	**0.940**	**0.971**	**0.943**
		CB-2	0.973

AVE square roots of latent constructs displayed in Table 3 showed them to be larger in comparison to the correlations of the corresponding latent-variable [2]. Meanwhile, values of HTMT ratio remained lower than 0.90 as recommended in literature [3,4] for discriminant validity confirmation.

**Table 3:** Discriminant validity based on Fornell-Larcker criterion and HTMT criterion.

Fornell-Larcker criterion							
Construct	1	2	3	4	5	6	7
Behavioral Intention to Cheat	0.892						
Cheating Behavior	0.696	0.971					
Opportunities	0.541	0.455	0.819				
Pressures	0.441	0.470	0.337	0.822			
Rationalization	0.475	0.364	0.487	0.374	0.737		
Social Norms	0.539	0.446	0.445	0.398	0.297	0.827	
Social Trust	0.566	0.506	0.376	0.332	0.394	0.419	0.832

HTMT criterion							

Construct	1	2	3	4	5	6	7

Behavioral Intention to Cheat	-						
Cheating Behavior	0.761	-					
Opportunities	0.664	0.539	-				
Pressures	0.521	0.532	0.419	-			
Rationalization	0.596	0.431	0.650	0.497	-		
Social Norms	0.608	0.492	0.550	0.460	0.360	-	
Social Trust	0.662	0.573	0.477	0.396	0.497	0.500	-

## 2 Experimental Design, Materials and Methods

To reiterate, data was gathered via Microsoft forms, from the Jordanian university students, who were experienced in the E-learning system. The students were studying undergraduate and master degrees in accounting, accounting and commercial law, accounting and business law and accounting information system. The study respondents participated voluntarily and did not have to be incentivized to do so. They were selected using snowball sampling method because majority of similar studies also made use of the same approach. Three hundred and nineteen (231) students returned the questionnaires, but only 213 were fully completed and deemed useable. The sample size criteria of the study met the literature recommendation [7], and prior to the actual study, a pilot study was carried out to confirm the reliability of the questionnaire.

In the pilot study, 35 questionnaires were disseminated to students from the sample. The content and constructs validity of the questionnaire items were validated using the assistance of academic experts. Data analysis was conducted employing statistical test of PLS-SEM as it is the most suitable when the research objectives involve testing existing theories and complex model structures [8].

There are two stages of PLS-SEM analysis, namely measurement model specification and structural model evaluation [1]. The measurement model quality was also established by obtaining the items loadings (λ) of the constructs (see Table 2 and Fig. 1), which were found to be significant and over 0.70. For construct reliability, the study tested its presence using CR and Cronbach's alpha, which should be within 0.70 and 1.00 [9]. The results supported the presence of the reliability of constructs. The results are detailed in Table 2, which confirms the internal consistency of the measurement model.

Moving on to discriminant validity, researchers employ two criteria, namely Fornell-Larcker and HTMT ratio. Specifically, Fornell-Larcker criterion is considered to be a traditionally method and the results should show that AVE square root is higher than the correlation values between it and the rest of the constructs [2]. Meanwhile, HTMT is a relatively modern approach for discriminant validity evaluation, and the value has to be lower than 0.90 to establish sufficient discriminant validity [1]. Construct reliability and discriminant validity results are displayed in Table 3.

## Ethics Statement

The ethical approval has been obtained from Irbid National University; the ethical protocol number is 11/109. Informed consent was obtained from all participant in this study. The authors kept the ethical concerns into consideration when gathering data and ensured that the information obtained from the respondents were only utilized for research purposes, and is kept confidential and private at all times. In addition, data in this paper have not been acquired in violation of applicable law or by using human or animal subjects.

## CRediT authorship contribution statement

**Mohannad Obeid Al Shbail:** Supervision, Software, Validation, Writing – original draft, Formal analysis. **Hashem Alshurafat:** Investigation, Writing – review & editing, Resources. **Husam Ananzeh:** Conceptualization, Data curation. **Jebreel Mohammad Al-Msiedeen:** Conceptualization, Visualization.

## Declaration of Competing Interest

The research group was not a recipient of any financial support from any entity or institution and the authors have no known competing financial interests or personal relationships that could have affected the work presented by the paper.
